# Supplementary material for: Crystal structure of Drosophila Piwi
Source: Nat Commun. 2020 Feb 12;11:858. doi: 10.1038/s41467-020-14687-1 (PMC7015924; doi:10.1038/s41467-020-14687-1)
Supplement: Supplementary file 1 — Supplementary Information [file 41467_2020_14687_MOESM1_ESM.pdf]

Supplementary Information

Crystal structure of *Drosophila* Piwi

Yamaguchi, Oe, Nishida, Yamashita *et al.*

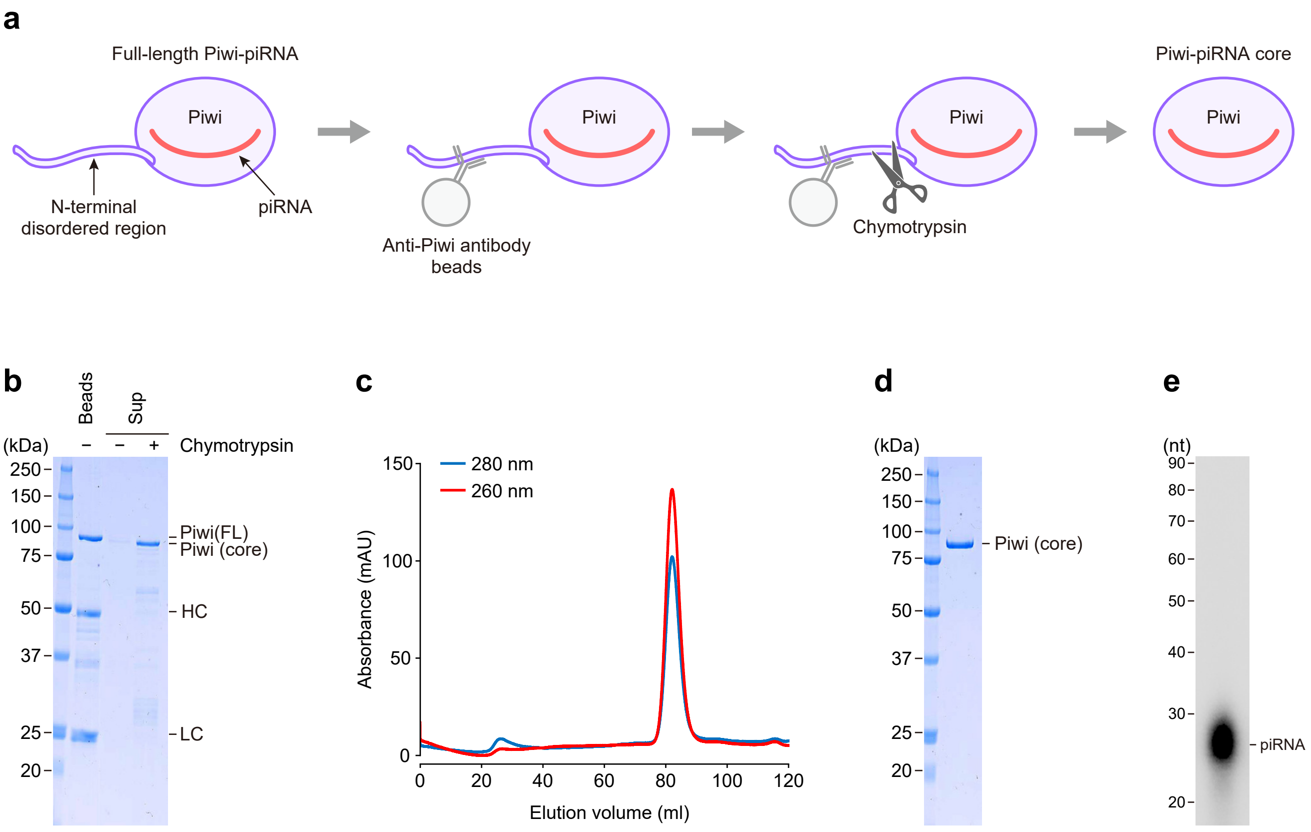

Supplementary Figure 1 | Preparation of the Piwi-piRNA complex.

a Purification scheme of the endogenous Piwi-piRNA complex from OSCs.

b Separation of the Piwi-piRNA complex from the anti-Piwi antibody beads. The full-length (FL) Piwi bound to the antibody beads was incubated in the absence and presence of chymotrypsin at 4°C overnight, and the supernatants were then analyzed by SDS-PAGE. The gel was stained with SimplyBlue SafeStain (Thermo Fisher Scientific). HC, heavy chain; LC, light chain.

c Elution profile of the purified Piwi-piRNA complex from the HiLoad 16/600 Superdex 200 column.

d SDS-PAGE analysis of the purified Piwi-piRNA complex. The gel was stained with SimplyBlue SafeStain.

e Denaturing urea-PAGE of the purified Piwi-piRNA complex. The bound piRNA was 5′ ^{32}P-labeled.

Source data are provided as a Source Data file.

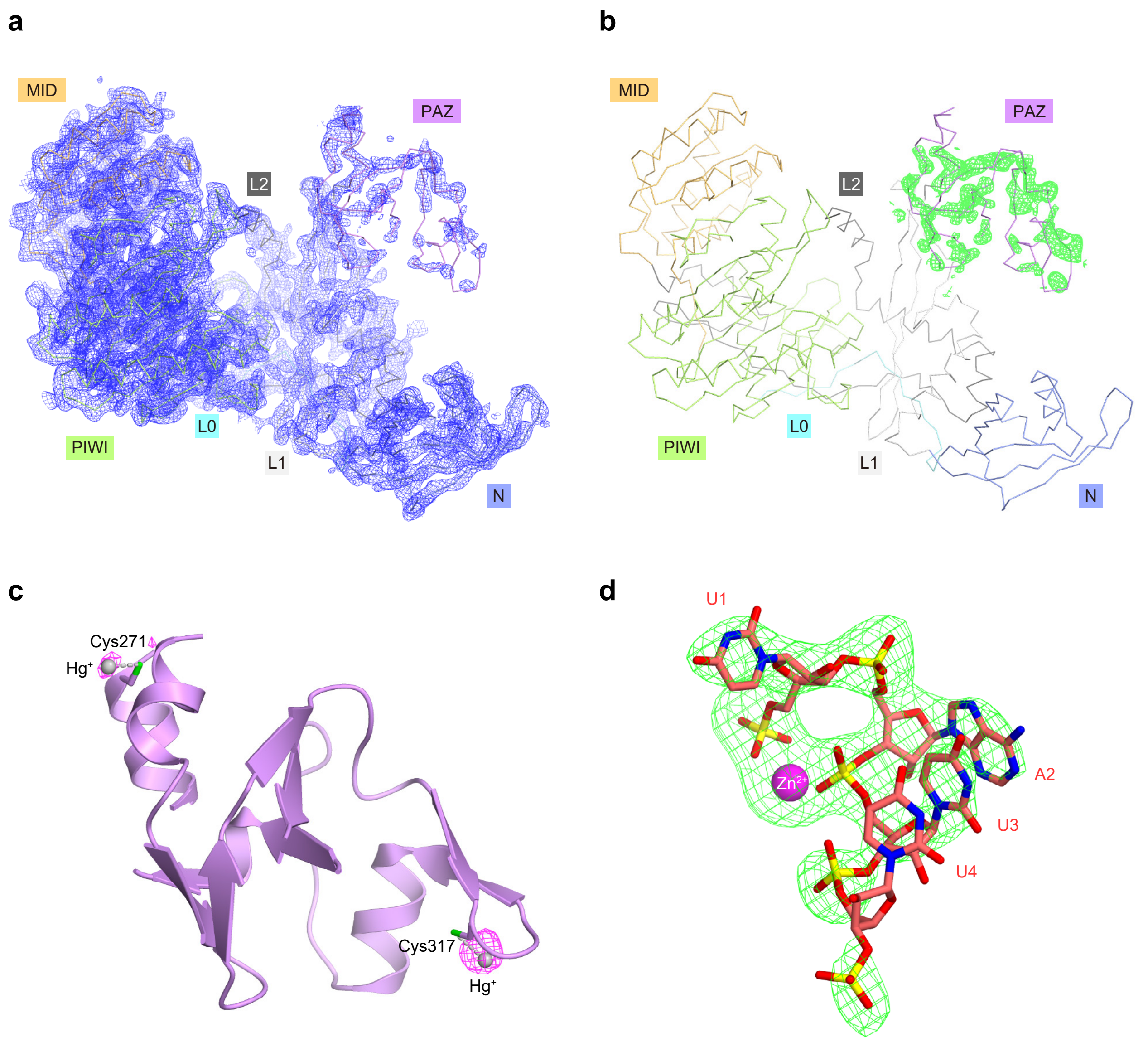

Supplementary Figure 2 | Electron density map.
a $2mF_o - DF_c$ electron density map for the Piwi-piRNA complex (contoured at 1σ).
b $mF_o - DF_c$ omit electron density map for the PAZ domain (contoured at 2σ).
c Anomalous difference density map of the mercury ions bound to Cys271 and Cys317 in the PAZ domain (contoured at 3.5σ).
d $mF_o - DF_c$ omit electron density map for the piRNA and the zinc ion (contoured at 3σ).

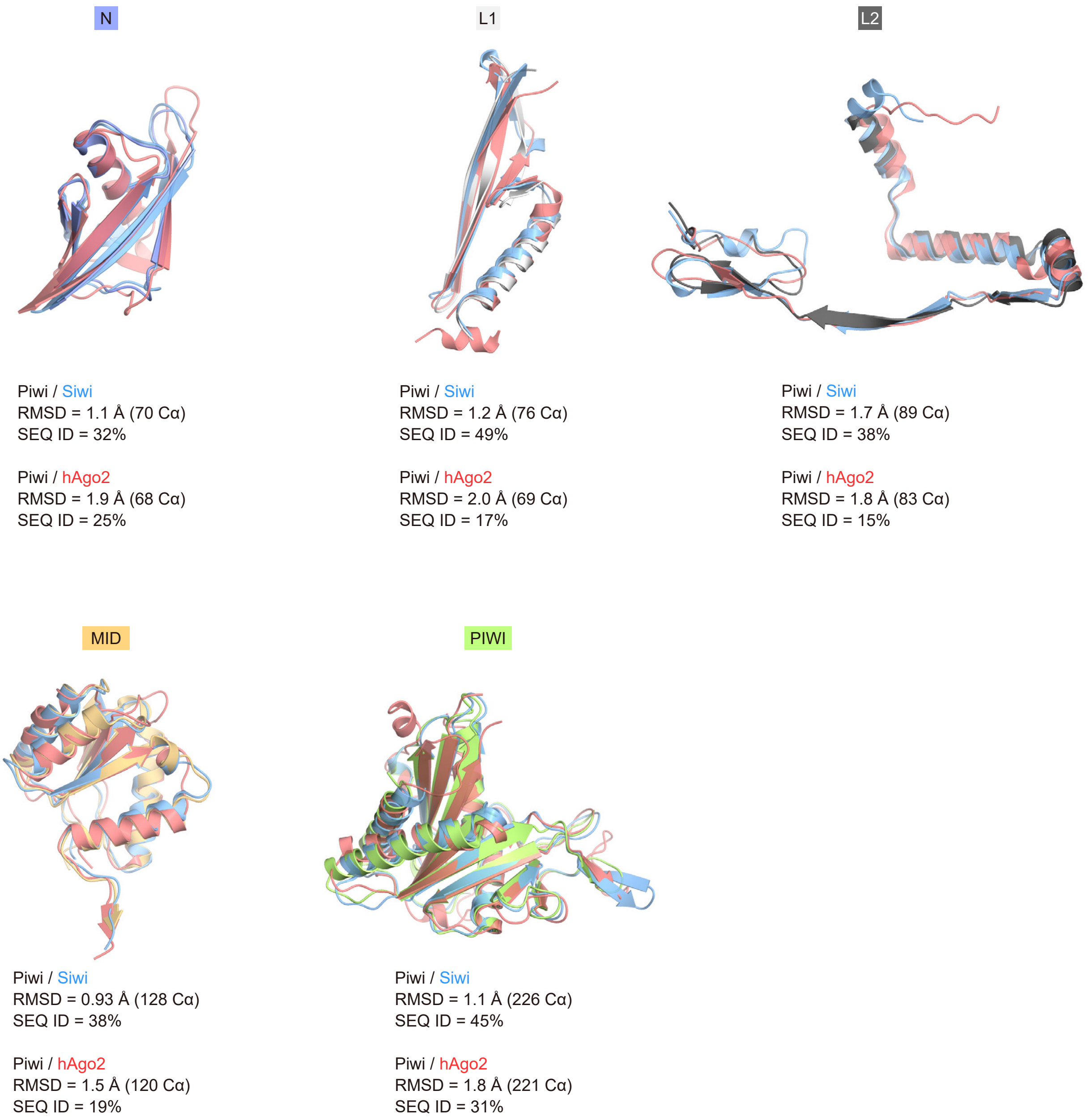

Supplementary Figure 3 | Structural comparison of Piwi with Siwi and hAgo2.
Superimpositions of the individual domains of Piwi, Siwi (PDB: 5GUH) (blue), and hAgo2 (PDB: 4W5N) (red). Sequence identities and root mean square deviation (RMSD) values for equivalent Cα atoms are shown below the structures.

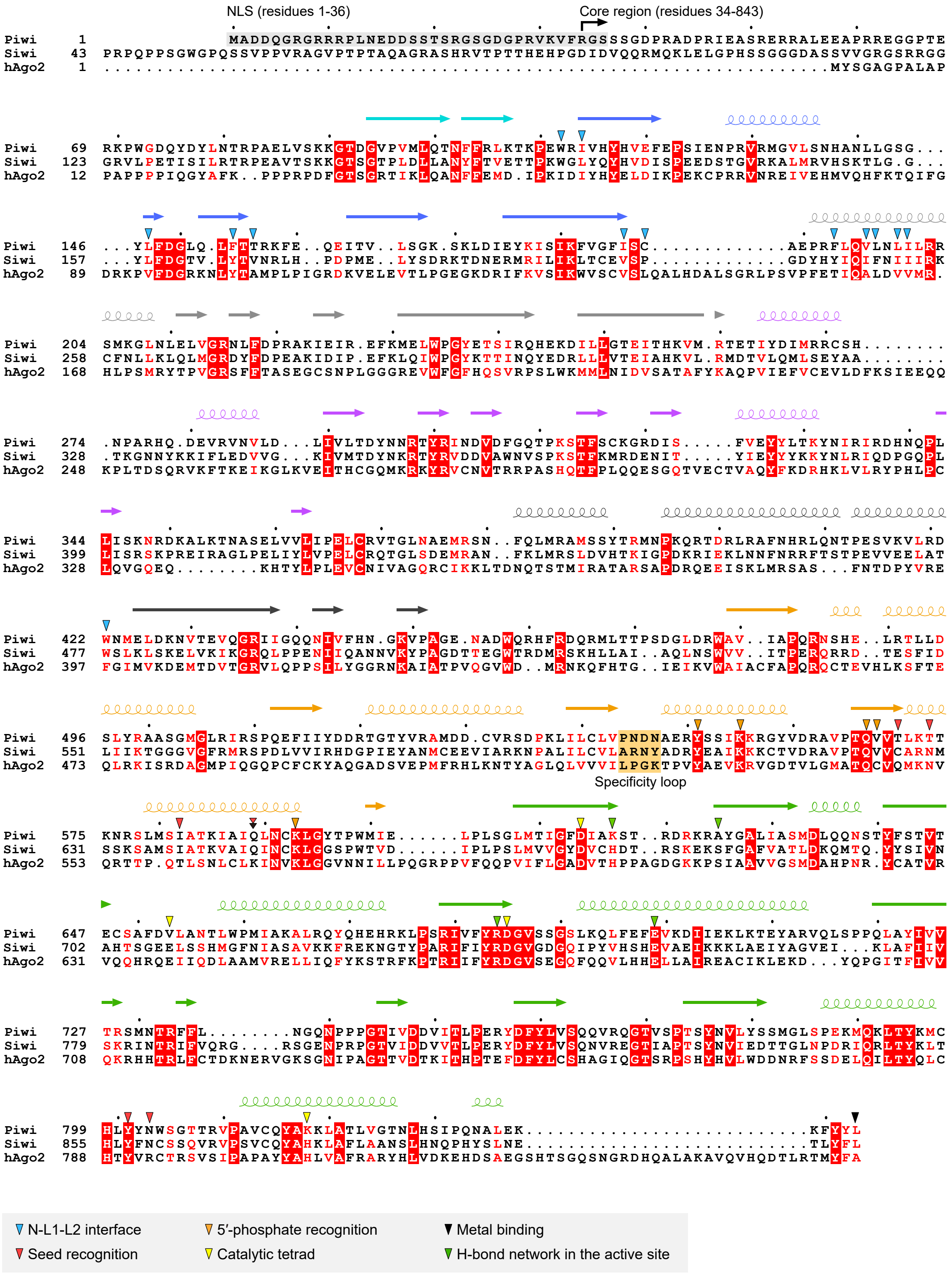

Supplementary Figure 4 | Structure-guided sequence alignment of Piwi, Siwi, and hAgo2.
The secondary structure of Piwi is indicated above the sequences. The specificity loop is highlighted in orange, and key residues are marked by triangles. The figure was prepared using Clustal Omega (http://www.ebi.ac.uk/Tools/msa/clustalo) and ESPript3 (http://espript.ibcp.fr/ESPript/ESPript).

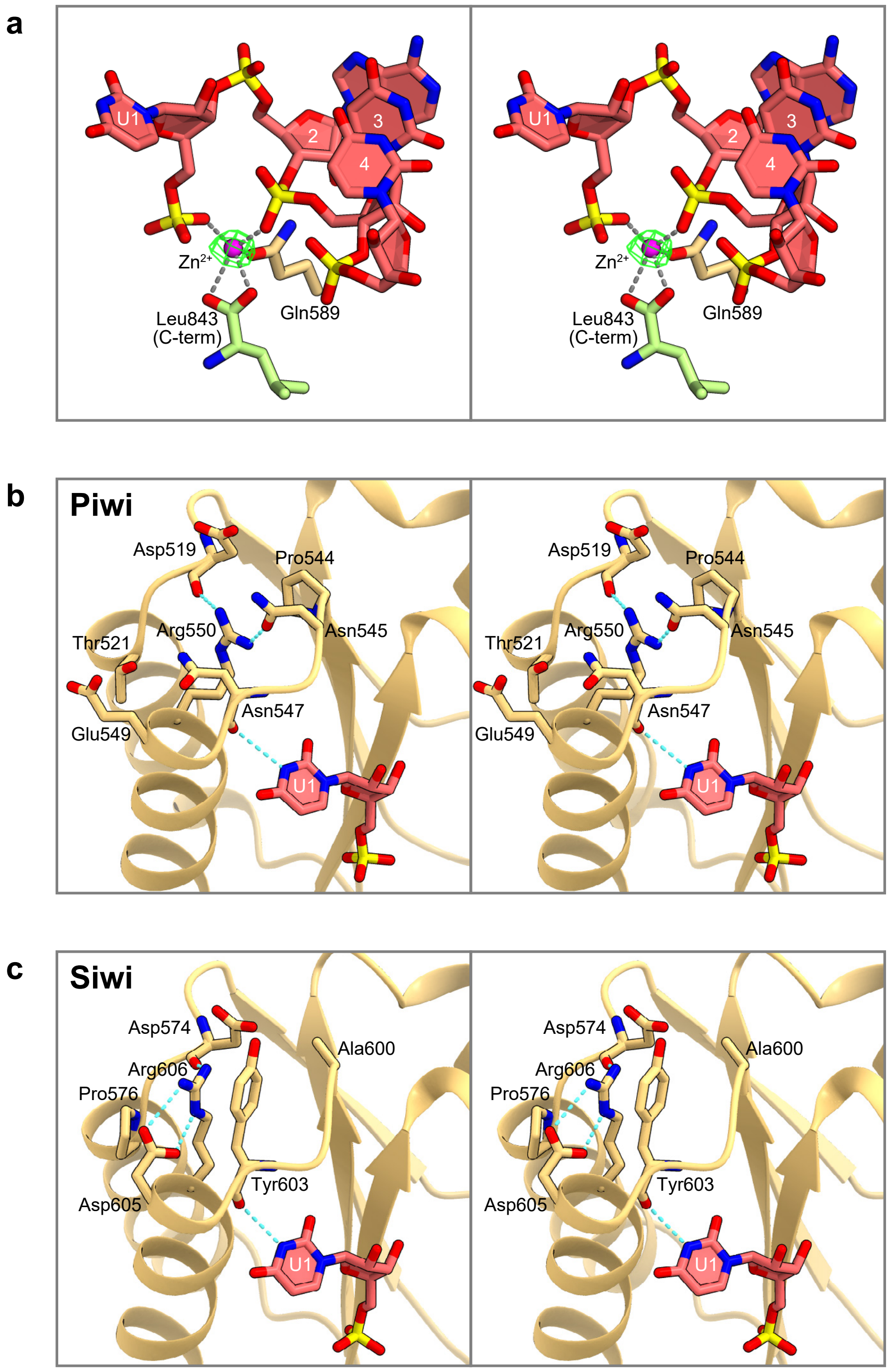

Supplementary Figure 5 | Recognition of the piRNA 5′ end.
a Anomalous difference density map for the bound zinc ion (contoured at 7σ) (stereo view).
b, **c** Specificity loops of Piwi (**b**) and Siwi (**c**) (stereo view).

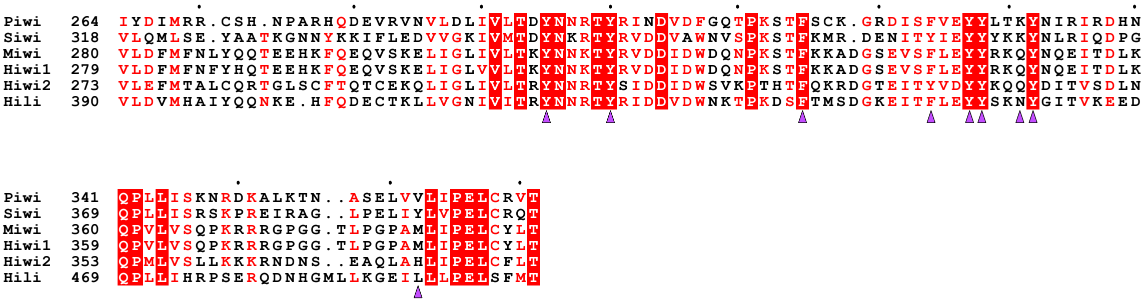

Supplementary Figure 6 | Sequence alignment of the PIWI PAZ domains.

The residues interacting with the piRNA 3′ end are marked by triangles. The figure was prepared using Clustal Omega and ESPript3.

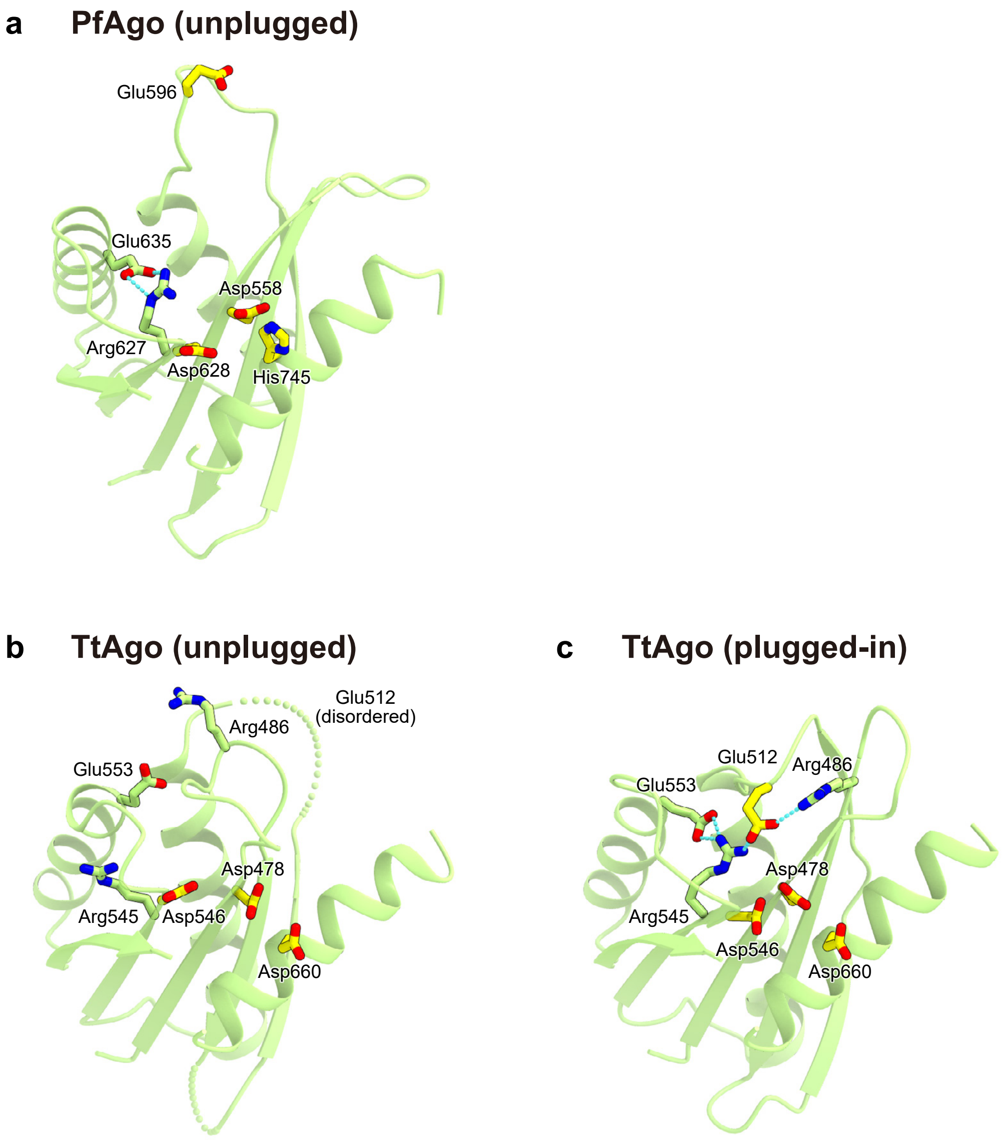

Supplementary Figure 7 | Catalytic tetrad of the prokaryotic AGOs.
a–**c** Catalytic tetrads of PfAgo in the apo state (PDB: 1U04) (**a**), and TtAgo in the guide-bound state (PDB: 3DLH) (**b**) and the guide-target-bound state (PDB: 4NCB) (**c**).

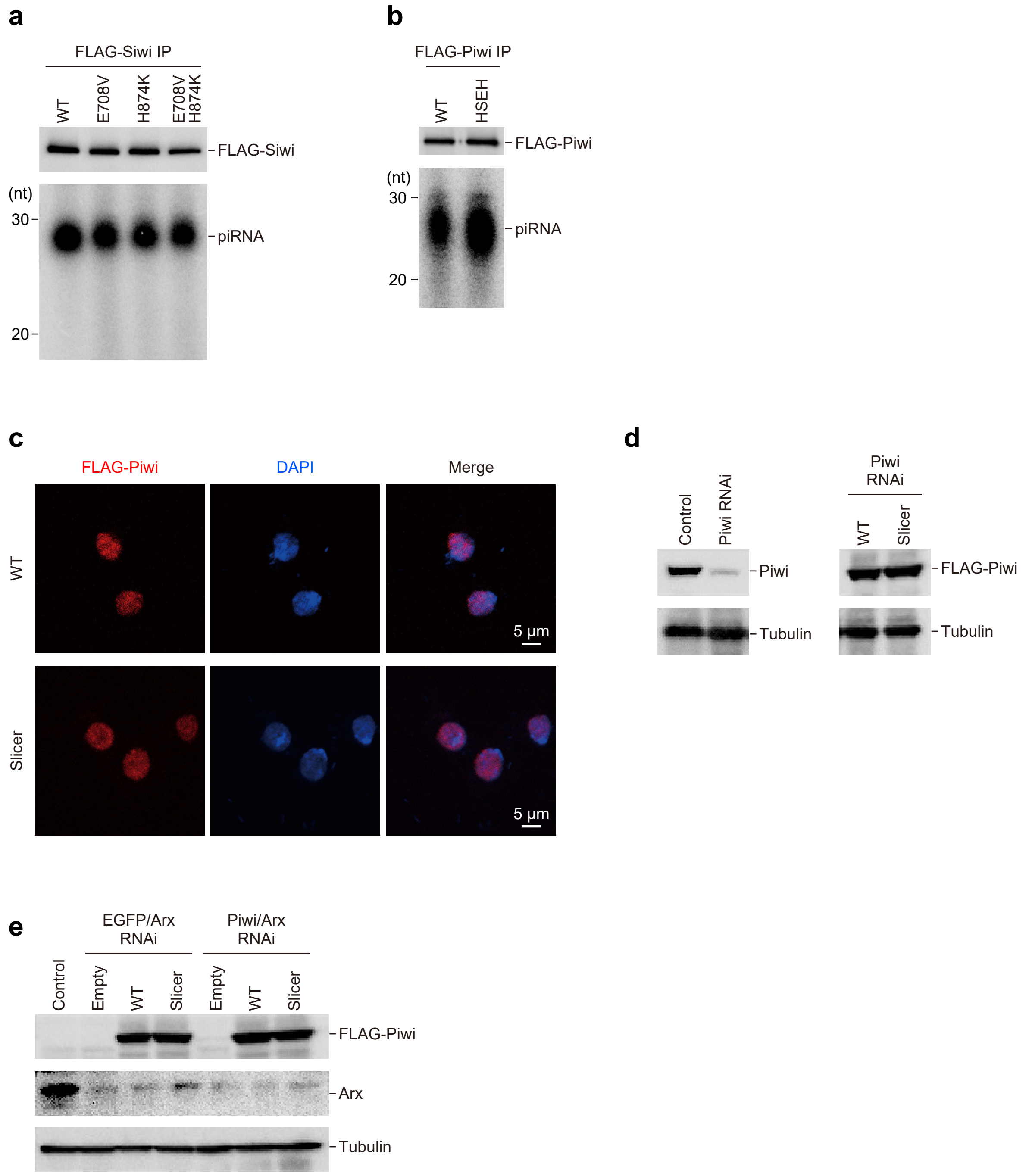

Supplementary Figure 8 | Expression of Piwi.

a Western blots of the FLAG-tagged wild-type Siwi and mutants expressed in BmN4 cells, and denaturing urea-PAGE of the ^{32}P-labeled bound piRNAs.

b Western blots of the FLAG-tagged wild-type Piwi and slicer-Piwi expressed in OSCs, and denaturing urea-PAGE of the ^{32}P-labeled bound piRNAs. HSEH, the slicer-Piwi K617H/A625S/V653E/K818H mutant.

c Localization of FLAG-tagged wild-type Piwi and slicer-Piwi in OSCs. Slicer, the slicer-Piwi K617H/A625S/V653E/K818H mutant.

d Western blots of the endogenous Piwi, FLAG-tagged wild-type Piwi, and FLAG-tagged slicer-Piwi expressed in Piwi-depleted OSCs. Tubulin was used as a loading control.

e Western blots of the FLAG-tagged wild-type Piwi and slicer-Piwi expressed in Piwi/Arx-depleted OSCs. Empty, empty vector control.

Source data are provided as a Source Data file.

Supplementary Table 1 | Oligonucleotides used in this study

Gene	Primer	Sequence (5′ to 3′)
Piwi	K617H-F	CATAGCACACGAGATCGGAAGAGGG
	K617H-R	CGCAATGTCAAAGCCAATTGTCATC
	A625S-F	TCGGAAGAGGTCCTACGGAGCATTG
	A625S-R	TCTCGTGTGCTCTTCGCAATGTCAA
	V653E-F	AGCTCGCTAACACCCTTTGGCCGAT
	V653E-R	CATCAAAGGCGCTGCACTCCGTGAC
	K818H-F	CATAAGTTAGCTACACTCGTGGGTA
	K818H-R	AGCGTACTGGCAAACTGCTGGCACT
Siwi	E708V-F	TACTCAGCTCTCACATGGGTTTCAA
	E708V-R	CTTCTCCGGAAGTGTGCGCATTGAC
	H874K-F	AAGAAACTTGCATTCCTTGCAGCTA
	H874K-R	GGCATATTGGCAGACCGATGGCACG
flam	Target RNA-F	TAATACGACTCACTATAGGGCCCCCGTTAACTAAAACTGTCTGATCCTCATA
	Target RNA-R	GCCCCCCCTCGAGGTCGACGGTATCGATAAGATATACTATGAGGATCAGACAGTTTTAGT
Piwi PAZ	pESUMO-F	AAGCTTGCGGCCGCACTCGAGCACC
	pESUMO-R	ACCTCCAATCTGTTCGCGGTGAGCCTC
	PAZ-F	GAACAGATTGGAGGTGAGACGATCTACGACATAATGCGACGT
	PAZ-R	TGCGGCCGCAAGCTTTTAATTGAGCCCAGTCACTCGGCAG
