# Supplementary material for: Crystal structure of Drosophila Piwi
Source: Nat Commun. 2020 Feb 12;11:858. doi: 10.1038/s41467-020-14687-1 (PMC7015924; doi:10.1038/s41467-020-14687-1)
Supplement: Supplementary file 3 — Reporting Summary [file 41467_2020_14687_MOESM3_ESM.pdf]

nature research

Corresponding author(s): Hiroshi Nishimasu, Mikiko C. Siomi, Osamu Nureki

Last updated by author(s): Jan 10, 2020

Reporting Summary

Nature Research wishes to improve the reproducibility of the work that we publish. This form provides structure for consistency and transparency in reporting. For further information on Nature Research policies, see Authors & Referees and the Editorial Policy Checklist .

Statistics

For all statistical analyses, confirm that the following items are present in the figure legend, table legend, main text, or Methods section.

n/a	Confirmed	
☐	☒	The exact sample size (*n*) for each experimental group/condition, given as a discrete number and unit of measurement
☐	☒	A statement on whether measurements were taken from distinct samples or whether the same sample was measured repeatedly
☒	☐	The statistical test(s) used AND whether they are one- or two-sided<br>*Only common tests should be described solely by name; describe more complex techniques in the Methods section.*
☒	☐	A description of all covariates tested
☒	☐	A description of any assumptions or corrections, such as tests of normality and adjustment for multiple comparisons
☐	☒	A full description of the statistical parameters including central tendency (e.g. means) or other basic estimates (e.g. regression coefficient) AND variation (e.g. standard deviation) or associated estimates of uncertainty (e.g. confidence intervals)
☒	☐	For null hypothesis testing, the test statistic (e.g. *F*, *t*, *r*) with confidence intervals, effect sizes, degrees of freedom and *P* value noted<br>*Give P values as exact values whenever suitable.*
☒	☐	For Bayesian analysis, information on the choice of priors and Markov chain Monte Carlo settings
☒	☐	For hierarchical and complex designs, identification of the appropriate level for tests and full reporting of outcomes
☒	☐	Estimates of effect sizes (e.g. Cohen's *d*, Pearson's *r*), indicating how they were calculated

Our web collection on statistics for biologists contains articles on many of the points above.

Software and code

Policy information about availability of computer code

Data collection	N/A
Data analysis	KAMO, DIALS, XSCALE, Phaser, Refmac5, Sculptor, COOT, Prosmart

For manuscripts utilizing custom algorithms or software that are central to the research but not yet described in published literature, software must be made available to editors/reviewers. We strongly encourage code deposition in a community repository (e.g. GitHub). See the Nature Research guidelines for submitting code & software for further information.

Data

Policy information about availability of data

All manuscripts must include a data availability statement. This statement should provide the following information, where applicable:

- Accession codes, unique identifiers, or web links for publicly available datasets
- A list of figures that have associated raw data
- A description of any restrictions on data availability

The atomic coordinates of the Piwi-piRNA complex have been deposited in the Protein Data Bank, with the accession number PDB: 6KR6.

Field-specific reporting

Please select the one below that is the best fit for your research. If you are not sure, read the appropriate sections before making your selection.

☒ Life sciences ☐ Behavioural & social sciences ☐ Ecological, evolutionary & environmental sciences

For a reference copy of the document with all sections, see nature.com/documents/nr-reporting-summary-flat.pdf

Life sciences study design

All studies must disclose on these points even when the disclosure is negative.

Sample size	No sample size calculation was done. Sample size was estimated to be sufficient based on the consistency and magnitude of measured differences between groups.
Data exclusions	No exclusion.
Replication	All measurements were repeated at least three times in biochemical assays. All attempts at replication were successful.
Randomization	No randomization.
Blinding	No blinding.

Reporting for specific materials, systems and methods

We require information from authors about some types of materials, experimental systems and methods used in many studies. Here, indicate whether each material, system or method listed is relevant to your study. If you are not sure if a list item applies to your research, read the appropriate section before selecting a response.

Materials & experimental systems

n/a	Involved in the study	
☐	☒	Antibodies
☐	☒	Eukaryotic cell lines
☒	☐	Palaeontology
☒	☐	Animals and other organisms
☒	☐	Human research participants
☒	☐	Clinical data

Methods

n/a	Involved in the study	
☒	☐	ChIP-seq
☒	☐	Flow cytometry
☒	☐	MRI-based neuroimaging

Antibodies

Antibodies used	Anti-Arx antibody, Anti-H3 antibody, Anti-Piwi monoclonal antibody 3G11, Alexa Fluor 555 goat anti-mouse immunoglobulin, Anti-DDDDK-tag mAb, Anti-FLAG antibody, Anti-Tubulin antibody
Validation	Anti-Arx antibodies were produced from immunized mouse. Anti-H3 antibodies, Anti-Piwi monoclonal antibodies 3G11, Alexa Fluor 555 goat anti-mouse immunoglobulin, Anti-DDDDK-tag (catalog number: FLA-1), Anti-FLAG antibodies and Anti-Tubulin antibodies (catalog number: E7) were purchased from Abcam, Mikuri Immunology Laboratory, Invitrogen, MBL, Sigma-Aldrich and DSHB, respectively.

Eukaryotic cell lines

Policy information about cell lines

Cell line source(s)	OSCs were developed from fGS/OSS line as previously described (Saito et al., 2009). BmN4 cells were gifted from National Institute of Agrobiological Sciences (NIAS).
Authentication	OSCs and BmN4 cells are both authenticated.
Mycoplasma contamination	OSCs and BmN4 cells were not tested for mycoplasma contamination.
Commonly misidentified lines (See ICLAC register)	OSCs and BmN4 cells are not misidentified cell lines.
